# Supplementary figures and images for: Glial-dependent clustering of voltage-gated ion channels in Drosophila precedes myelin formation
Source: eLife. 2023 Jun 6;12:e85752. doi: 10.7554/eLife.85752 (PMC10287160; doi:10.7554/eLife.85752)

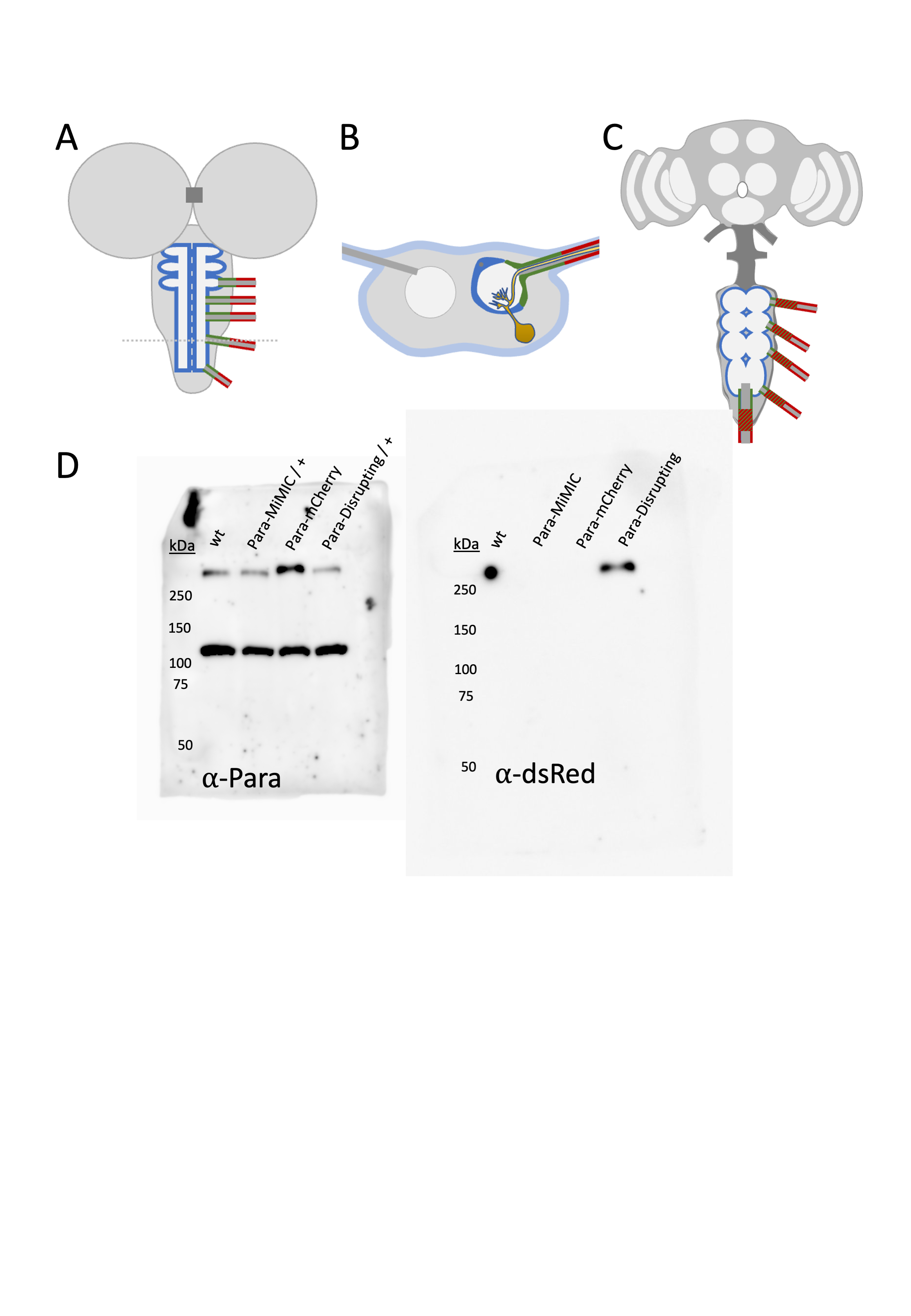

Supplement: Figure 1—figure supplement 1—source data 1. [file elife-85752-fig1-figsupp1-data1.zip › Sup Fig. 1 source data/Folie19.tiff]

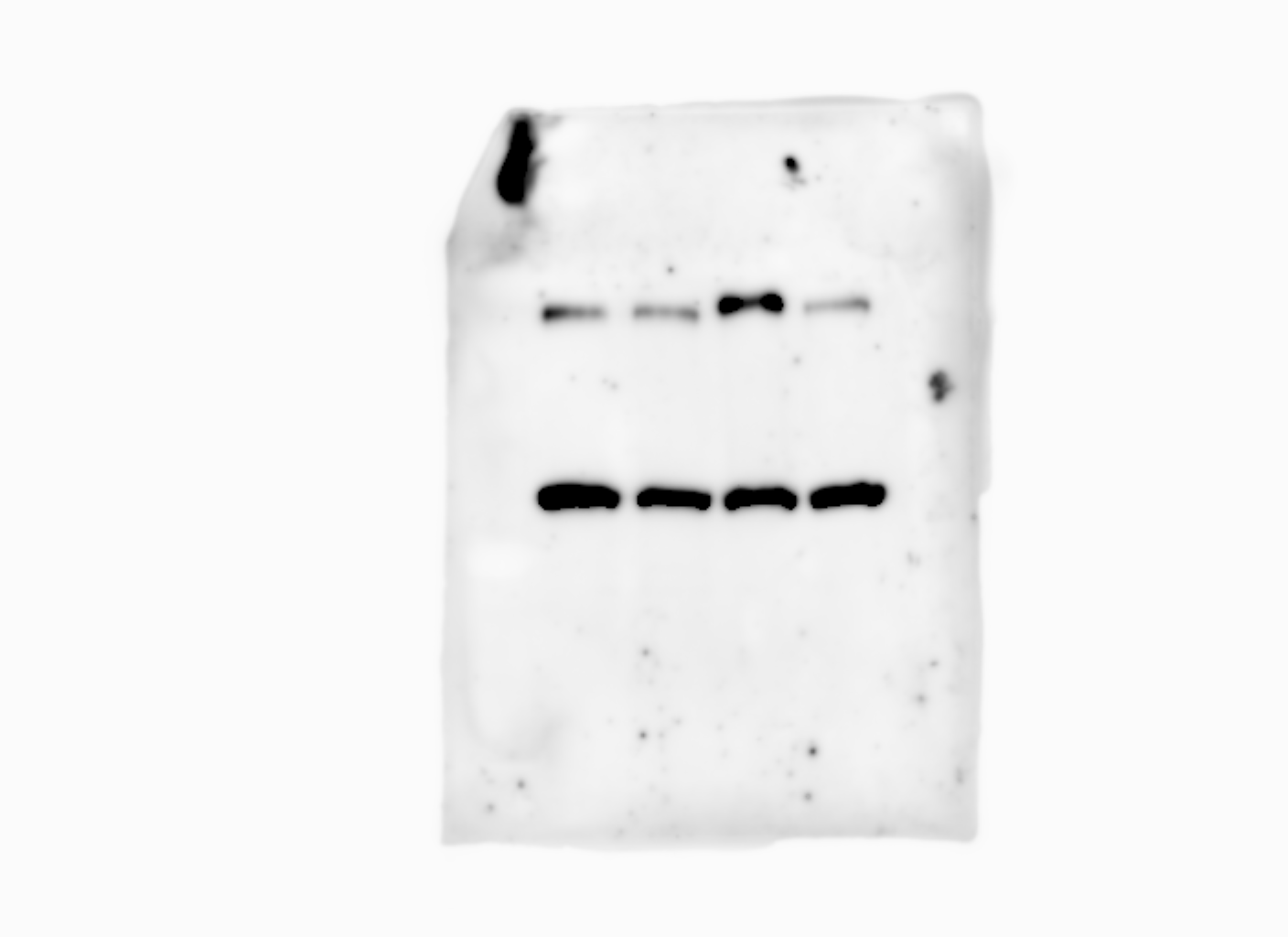

Supplement: Figure 1—figure supplement 1—source data 1. [file elife-85752-fig1-figsupp1-data1.zip › Sup Fig. 1 source data/20210401-Para Ab, wt, Mi, mCh, D 2 2021.04.01_11.28.07_Ch.tif]

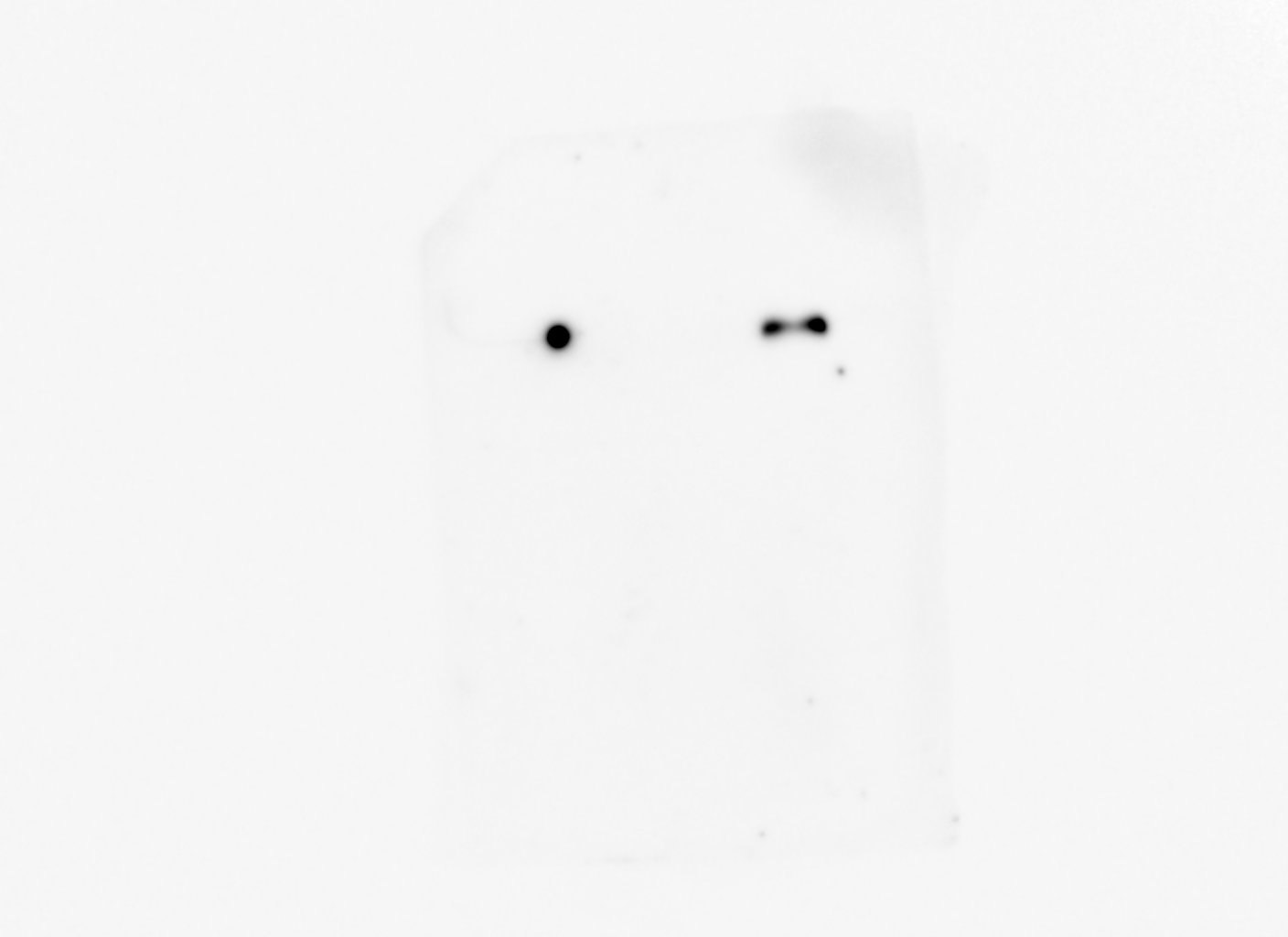

Supplement: Figure 1—figure supplement 1—source data 1. [file elife-85752-fig1-figsupp1-data1.zip › Sup Fig. 1 source data/20210401-dsRed Ab, wt, Mi, mCh, D 1 2021.04.01_11.33.05_Ch.jpg]

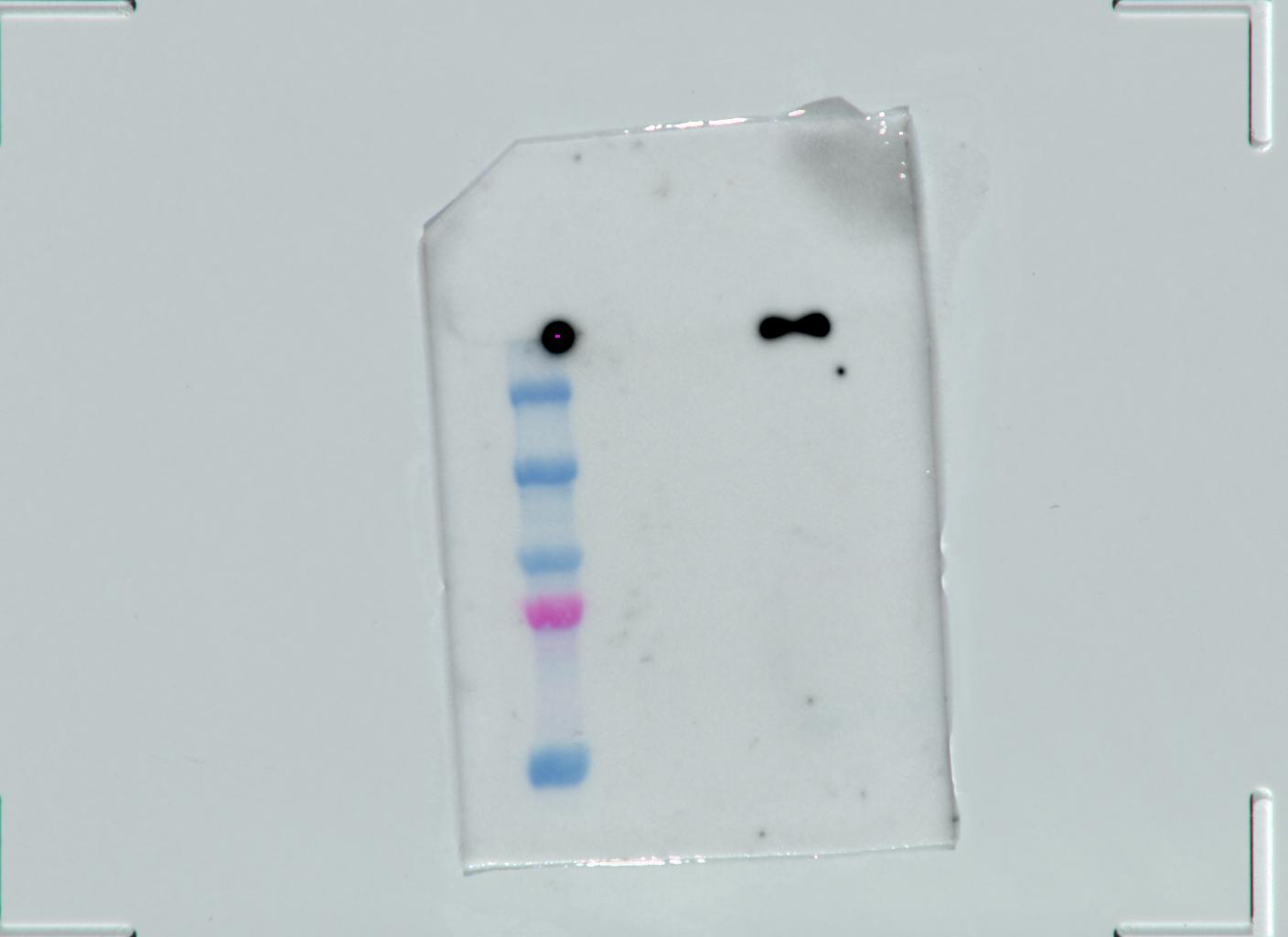

Supplement: Figure 1—figure supplement 1—source data 1. [file elife-85752-fig1-figsupp1-data1.zip › Sup Fig. 1 source data/20210401-dsRed Ab, wt, Mi, mCh, D 1 2021.04.01_11.33.05_Ch+Marker.jpg]

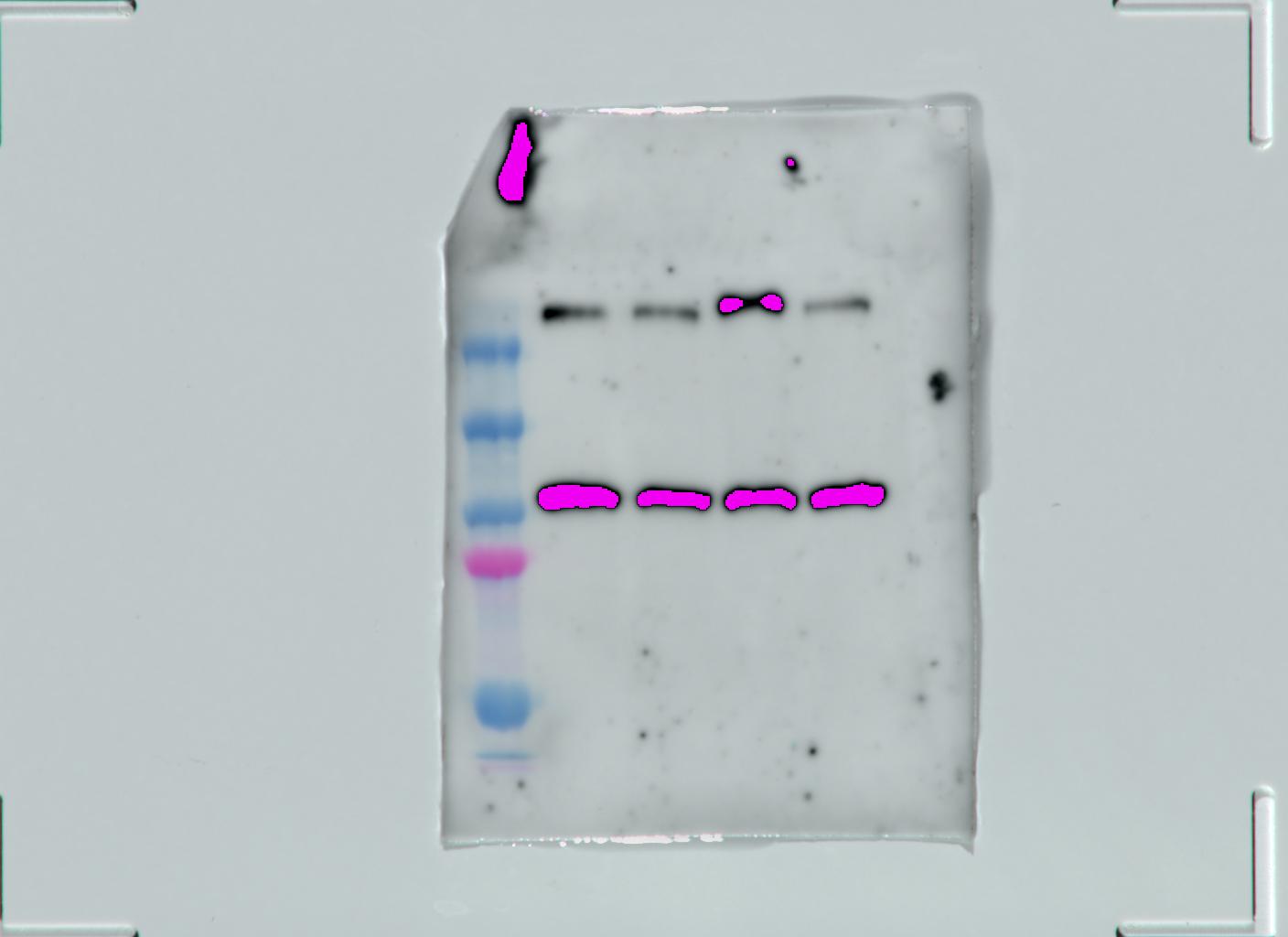

Supplement: Figure 1—figure supplement 1—source data 1. [file elife-85752-fig1-figsupp1-data1.zip › Sup Fig. 1 source data/20210401-Para Ab, wt, Mi, mCh, D 2 2021.04.01_11.28.07_Ch+Marker.jpg]
